# Putting biomonitors to work: native moss as a screening tool for solid waste incineration

**DOI:** 10.1007/s10661-024-13354-y

**Published:** 2024-11-07

**Authors:** Sarah Jovan, Eleonore Jacobson, Jason M. Unrine, Nasser Jalili-Jahani, Bruce McCune

**Affiliations:** 1 U.S. Forest Service, Pacific Northwest Research Station, Portland, OR USA; 2 Department of Botany and Plant Pathology, Oregon State University, Corvallis, OR 97331 USA; 3 Department of Plant and Soil Sciences, University of Kentucky, Lexington, KY 40546 USA; 4 University of Kentucky, Kentucky Water Research Institute, Lexington, KY 40546 USA

**Keywords:** Atmospheric deposition, Biomonitoring, Europium, Gadolinium, Heavy metals, Trace metals

## Abstract

Solid waste incineration (SWI) can release numerous air pollutants although the geographic reach of emissions is not routinely monitored. While many studies use moss and lichens for biomonitoring trace elements, including around SWIs, few investigate the complex, multi-element footprint expected from SWI emissions. This study develops using native moss as a screening tool for SWI while also informing community concerns about an aging incinerator in rural Oregon, USA. Trained community volunteers helped collect 36 composite samples of epiphytic moss (*Orthotrichum* s.l.) along a 32-km transect from the SWI. We used ICP-MS to measure 40 elements in moss, including 14 rare earth elements (REEs) previously unexplored for SWI. We compared the elemental signatures of samples with an emissions profile for SWI and modeled relationships between element concentrations and distance from the facility using nonparametric regression. The chemical signatures in moss pointed to SWI as a source, potentially through both stack and fugitive dust emissions. The strongest models described farther-dispersing elements, including mercury and cadmium (xR^2^ = 0.65 and 0.62, respectively), and suggested most deposition occurs within 5 to 10 km of the facility. Elements often associated with soil and dust, like arsenic and chromium, exhibited localized peaks within 0.2 km of the incinerator (xR^2^ = 0.14–0.3). Three novel elements—cesium and REEs europium and gadolinium—also showed promise as atmospheric tracers for SWI. Gadolinium, a contrast reagent for MRIs, could reflect medical waste incineration by the facility. We include additional analysis and discussion to help stakeholders use results effectively.

**Supplementary Information:**

The online version contains supplementary material available at 10.1007/s10661-024-13354-y.

## Introduction

Biomonitoring is a powerful scientific tool for investigating air quality. Although reference-grade air monitors provide the most accurate depiction of pollution levels, high costs prevent their widespread deployment, making it challenging to capture the spatial extent of air pollutants (WHO, 2023). To address this gap, hundreds of studies since the 1960s have utilized moss and lichens as biomonitors to investigate the composition and spatial distribution of trace elements (Abas, 2021; Chaudhuri & Roy, 2023). These organisms absorb their water and nutrients predominantly from the atmosphere, leading to the bioaccumulation of many air contaminants that are otherwise expensive to measure.

A key goal of biomonitoring is to inform environmental decision-making, though many studies remain within scientific circles (Boquete et al., 2017; Pirintsos & Loppi, 2008). Lack of communication with potential users seems to be a common problem, including explaining how findings fit within a broader inquiry or investigative process. Effort by the scientist to “operationalize” findings may make the difference because biomonitoring data do not directly translate to air quality measurements or the regulatory and health thresholds decision-makers use (Bargagli, 2016; Varela et al., 2023). When applied explicitly as a screening tool, however, data may indirectly influence air quality policy and behavior by optimizing the use of those critical resources (e.g., air monitors, funding, decision-maker time and attention; Chiapella et al., 2019; Donovan et al., 2016; Gatziolis et al., 2016; Jovan et al., 2022; Vuković et al., 2015).

We would expect that biomonitoring would be an insightful screening tool for solid waste incineration (SWI). While SWIs are globally common as an alternative to landfilling, their potential to emit numerous hazardous air pollutants (HAPs), including trace metals (Giusti, 2009; National Research Council, 2000), is controversial. The scientific community remains divided on the risks of living nearby, which are difficult to assess because each incinerator’s emissions are unique (Bolan et al., 2023; de Titto & Savino, 2019; Domingo et al., 2020; Tait et al., 2020) yet their dispersal into the surrounding environment is not routinely monitored (National Research Council, 2000).

Mandates typically require monitoring emissions on-site, which is sometimes used in computational models to predict their off-site dispersal. However, this approach relies on many assumptions, leading to inaccuracies compared to empirical methods (Gronwald & Chang, 2018; Holmes & Morawska, 2006), including biomonitoring screening datasets (Donovan et al., 2016; Gatziolis et al., 2016). For example, it is assumed that on-site measurements represent emissions even though measurements are taken periodically, even annually (National Research Council, 2000). Characterizing SWI emissions is challenging because they vary widely depending on the operating conditions and composition of materials being incinerated at the time of measurement (National Research Council, 2000). Transitioning from periodic to continuous monitoring of trace metals is discussed in the current study, although it is not yet the convention.

Several biomonitoring studies address SWI emissions (e.g., Antisari et al., 2011; Contardo et al., 2018; Protano et al., 2015; Tretiach et al., 2011), although to the best of our knowledge, only one captured a multi-element signature clearly indicating SWI (Loppi et al., 2000). Hypothetically, if emissions exceed background levels, we would expect biomonitors to capture a complex elemental signature reflecting the chemical heterogeneity of incinerated materials as well as the contrasting spatial scales of potential emissions pathways—i.e., via tall stacks meant to dilute and spread pollutants across large areas or else concentrated in the residual ash, which must be properly contained to prevent emissions as fugitive dust until disposal in a landfill (Bolan et al., 2023). The low cost of biomonitors allows for measuring numerous contaminants across many samples, as would be needed to describe a complex footprint. Additionally, native (i.e., in-situ) biomonitors reflect pollutant deposition over extended periods, ranging from months to years (Boquete et al., 2013; Kularatne & de Freitas, 2013; Paoli et al., 2018), and may therefore capture the episodic emissions that traditional air monitoring can miss (e.g., Donovan et al., 2016). While relationships are not 1:1, these data may provide information on the long-term exposure of residents in the vicinity of the incinerator.

The main goals of this study were to investigate native moss as a screening tool for SWI while also informing air quality concerns raised by communities near the facility in rural Oregon, USA. In addition to its older age, the facility is contentious for burning some medical waste, known to release higher HAPs (Bolan et al., 2023), despite being regulated by the weaker emissions standards set for municipal (vs. medical) waste incinerators. With the help of trained community volunteers, we sampled the tufted, epiphytic moss species abundant in our study region (mainly *Orthotrichum* spp.) along a 32-km transect extending from the incinerator. We measured 40 elements in each sample and compared their elemental signatures against an emissions profile for medical waste incinerators (Bolan et al., 2023). Our study is unique among incinerator studies for assessing numerous rare earth elements (REE) along with traditional trace metals. Despite increasing use in the medical and high-tech industries (Sager & Wiche, 2024; Urošević et al., 2020), REE emissions from SWI are unexplored. We expected results would provide an initial, empirical basis for discussing community concerns, and potentially also inform the implementation of new state-level regulations for SWI emissions. To help facilitate the understanding and use of results, we included targeted discussion for stakeholders alongside scientific reporting.

## Materials and methods

**Fig. 1:**
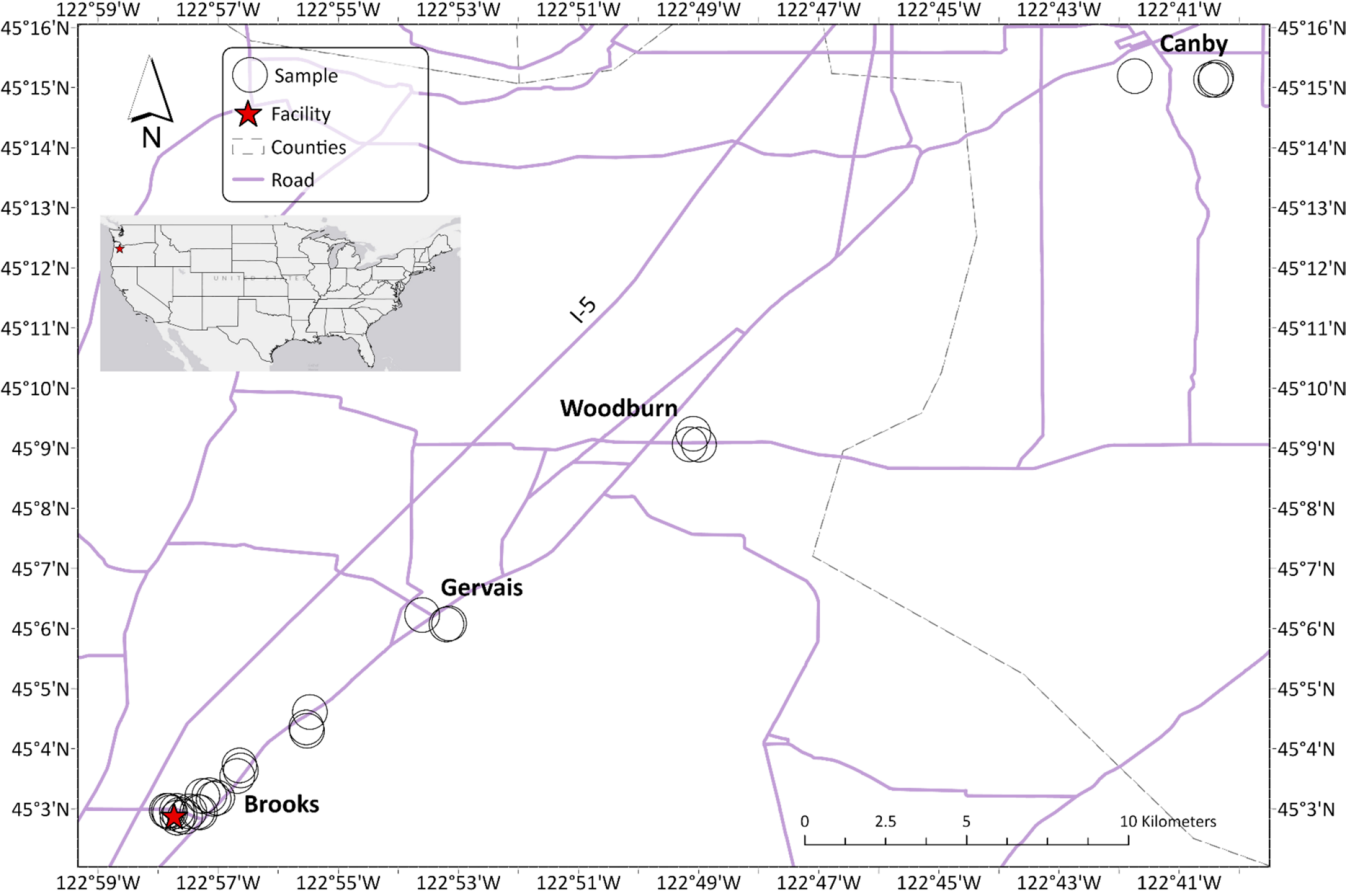
Map of the study area showing sample sites with major cities and the Interstate (I-5) labeled. Inset map shows location of the study area in the USA

The study area spanned parts of Marion and Clackamas Counties within the Willamette Valley of Oregon, USA (Fig. 1). The ecoregion is highly agricultural and rural but also contains most of Oregon’s population in scattered cities and townships, becoming densest in the Portland metropolitan area 20 km north of the study area. Major habitat types include wetlands, oak woodlands, and savannas. The temperate climate has cool wet winters and warm dry summers. Mean annual temperatures historically ranges from 10 to 13 °C, with precipitation annual range 89–160 cm, falling mostly in the winter as rain (Wiken et al., 2011).

The facility has been in continuous operation since 1986 in the small unincorporated community of Brooks, Oregon, surrounded mostly by residential and agricultural land as shown in Online Resource 1. The facility is classified as a municipal waste incinerator although some medical waste is incinerated each year. This study may inform three ongoing regulatory matters discussed in stakeholder-focused sections: (1) implementing new requirements for continuous emissions monitoring of certain contaminants under Senate Bill 488 (SB-488; 82nd Oregon Legislative Assembly, 2023), (2) renewal of the facility’s Title V permit to emit HAPs, and (3) compilation of an emissions inventory for the facility, a newer requirement under the Cleaner Air Oregon (CAO) Program that was created in response to a prior moss screening study (Chiapella et al., 2019; Donovan et al., 2016).

### Moss sampling

In August 2022, we collected moss samples (*n* = 36) along a NE transect at distances of 0.5, 1, 2, 4, 8, 16, and 32 km from the incinerator (Fig. 1). Ten samples were collected within 0.5 km and three per other listed distances at least 50 m apart, with six additional replicates spanning across the transect. The transect was oriented to the NE to align with the dominant W to SW winds (Online Resource 1) and did not intersect any major known sources of metals besides the incinerator.

Four adult community volunteers were trained to assist with moss sampling and processing. We used a protocol similar to a Seattle screening study where trained youth and adult community members led moss sampling and successfully produced a scientifically viable dataset (Derrien et al., 2020; Jovan et al., 2022), although the current study followed more rigorous sample handling and processing guidelines, described in Online Resource 2. Protocols otherwise followed Gatziolis et al. (2016). Briefly, eight or more subsamples of the moss *Orthotrichum* (broad sense, including *Pulvigera pringlei* and *P*. *papillosa*, formerly considered *O*. *lyellii,* along with *Lewinskia* spp*.*) were collected from varied locations on a single tree and analyzed as a composite sample for trace elements. These species have a similar acrocarpous growth form and frequently grow together. We periodically collected replicate samples to evaluate repeat-measurement errors. Samples were transported in a cooler with icepacks and stored at 4 °C until further processing. Moist samples were dried under a HEPA-filtered laminar flow, and any visual debris was removed after discarding the bottom third of the moss tufts.

### Trace element analysis

We used a review of medical waste incinerators to define the emissions profile for SWI, noting that municipal waste can release the same metals (Bolan et al., 2023): Silver (Ag), arsenic (As), barium (Ba), cadmium (Cd), cobalt (Co), chromium (Cr), copper (Cu), iron (Fe), mercury (Hg), manganese (Mn), molybdenum (Mo), nickel (Ni), lead (Pb), antimony (Sb), and zinc (Zn). Research suggests mosses are not reliable for biomonitoring Mn (Boquete et al., 2011) and potentially for Ba in *Orthotrichum* spp. (pers. obs) so we retained these as reference elements in the analysis to compare with those exhibiting spatial associations. We also included aluminum (Al), often used as a reference element to differentiate between atmospheric versus edaphic sources (i.e., soil or windblown dust) of trace elements measured in biomonitors (Giráldez et al., 2021).

We measured 23 additional elements that are relatively unexplored with respect to SWI. These include selenium (Se), the only element named in SB-488 that was not in the emissions profile (82nd Oregon Legislative Assembly, 2023). We also measured beryllium (Be), gallium (Ga), rubidium (Rb), strontium (Sr), thallium (Tl), uranium (U), vanadium (V), and 14 REEs: cerium (Ce), dysprosium (Dy), erbium (Er), europium (Eu), gadolinium (Gd), holmium (Ho), lanthanum (La), lutetium (Lu), neodymium (Nd), praseodymium (Pr), samarium (Sm), thallium (Tl), thulium (Tm), and ytterbium (Yb).

### Lab analysis and QA/QC

Moss samples were digested according to U.S. Environmental Protection Agency (EPA) method 3052 (EPA, 1996). Briefly, samples were first dried to a constant weight at 60 °C. Samples were then accurately weighed (0.250 g) and digested in 10 mL of trace-metal grade HNO_3_ (Aristar Plus, VWR, Radnor, PA, USA) in acid-cleaned, sealed polytetrafluoroethylene (PTFE) digestion vessels using a MARS 6 microwave digestion system (CEM, Matthews, NC, USA). The temperature was ramped from room temperature to 180 °C over 30 min and held at 180 °C for 15 min. Each set of up to 40 samples included digestion blanks and standard reference materials (SRM 1515- apple Leaves and SRM 1573a—tomato leaves, National Institute of Standards and Technology, Gaithersburg, MD, USA). The resulting digestates were clear and brought to 50 mL with 18 MΩ deionized water (DI water). Samples were then diluted 20 × with DI water for analysis of Fe, Mn and Al, and 4 × with DI water for all other elements. Samples were then analyzed by inductively coupled plasma mass spectrometry (ICP-MS, 7900, Agilent Technologies, Santa Clara, CA, USA) using U.S. EPA method 6020B (EPA, 2014). Matrix-matched external standards were prepared using certified reference materials (Inorganic Ventures, Blacksburg, VA, USA). An internal standard mix (Ge, Y, In, Tb and Bi) was added at 10 µg/L for all samples and standards. Quality control procedures included initial calibration verification (ICV), continuing calibration verification (CCV), initial blank verification (IBV), continuing blank verification (CBV), analysis of digestion replicates, analysis of dilution replicates, determination of spike recovery, analysis of standard reference materials (SRMs), and analysis of digestion blanks with each analytical batch (Online Resource 3). An octopole reaction system was used to suppress polyatomic interferences in H_2_ mode for Fe and Se and in He mode for Cr, Co, Ni, Cu and Zn. All other elements were analyzed in no-gas mode. Analyses of alternate isotopes and SRM recoveries were used to evaluate interferences.

### Detection limits

When fewer than 20% of the observations per element were below the detection limit, half the method detection limit was substituted for those values (Online Resource 4). Elements were excluded from further analysis if more than 20% of observations fell below the detection limit. Excluded elements were Lu, Ho, Th, B, and Ag.

### Statistical analysis

We used nonparametric regression (NPR) to relate elemental content of the moss to position along the transect from the incinerator. This method assumes no functional form and is thus able to capture complex nonlinear response surfaces (Bowman & Azzalini, 1997; McCune, 2006). The method is based on a kernel smoother, is fully nonparametric, and approximates the maximum likelihood solution, subject to penalization by leave-one-out cross validation in the model selection process. Model selection selects smoothing parameters by iterative trial-and-error to maximize × *R*^2^ (equivalent to a traditional *R*^2^ but penalized by cross validation). After transforming the data as log(elemental content) and log(distance from incinerator), we regressed each element against distance. We used a local linear model, Gaussian kernel, and the “medium” setting for overfitting controls in the software HyperNiche v. 2.30 (McCune & Mefford, 2009). Model fit was expressed with × *R*^2^.

### Correction for geogenic sources

We also performed similar regressions after adjusting elemental content relative to Al content, because normalization to Al content is commonly used to correct for soil-based particulates (Giráldez et al., 2021). Due to the modest sample size in this study (*n* = 36), we explored this analysis in several test cases but did not apply corrections across the study dataset. Residuals from the simple NPR of log(element Y) against log(Al) were saved as new Al-adjusted variables, then regressed against log(distance), again maximizing × *R*^2^ (penalizing by leave-one-out cross validation). Correction for Al content for elements with very weak relationships to Al would not have changed the results substantially, so we used residuals from regressions on Al for only those elements meeting two criteria: the cross-validated fit with Al was fairly strong (*R*^2^ > 0.30) and the element had some apparent declining relationship to distance from the incinerator. This resulted in a selection of eight elements: As, Ce, Co, Cr, Cs, Cu, Ni, and U.

We also tried adjusting REEs relative to Ce content, as the ratio of REEs to Ce in soils is fairly consistent globally (air) (Von der Kammer et al. 2012). If the relative abundance of a REE is much higher than the natural geogenic ratio, then it might suggest an anthropogenic source. In this case, however, we used residuals from linear fits of log(element Y) to log(Ce), because the relationships were so strongly linear.

### Additional analysis for stakeholders

We mapped eight “priority” elements based on their toxicity and use in former screening studies (As, Cd, Cr, Co, Hg, Mo, Ni, and Pb). Several of these had NPR models with inflection points around 1 km on the transect, so we grouped samples into nearer (< 1 km) and farther (≥ 1 km) sites in box plots. As reference points, we included the 95th percentiles from a screening study conducted in the nearby Portland metropolitan area (Gatziolis et al., 2016). That study was a systematic city-wide survey, so we use the 95th percentiles as context for discerning extreme values in datasets collected in a more targeted way (e.g., Jovan et al., 2022), as in the current study. We provide our full dataset to support additional analyses in Online Resource 4.

## Results

### Elemental gradients with distance

Moss concentrations of most elements in the emissions profile correlated with distance from the facility (Table 1). The profile elements most reported for incinerators, Cd, Hg, and Pb, had the strongest NPR models in this study (× *R*^2^ = 0.56–0.65). The next strongest models were for Cr, Cu, Mo, Ni, Sb, and Zn (× *R*^2^ = 0.29–0.53), followed by As and Co (× *R*^2^ = 0.15 for both). Model fits fell along a continuum, and we considered elements with any poorer fits too weak to discuss further. This included Se, an element listed in SB-488 (× *R*^2^ = 0.09), and the two profile elements we included for reference, Mn and Ba (× *R*^2^ =  − 0.11 and − 0.07, respectively; Table 1).

**Table 1:** Incinerator element-distance models and ranges. Models are nonparametric regression (local linear) of log(element) content of mosses against log(distance) from the incinerator; × *R*^2^ = leave-one-out cross-validated *R*^2^; ave N* = average neighborhood size for estimate; tolerance = smoothing parameter (s.d. of Gaussian weighting function for kernel smoother) in distance units (log(km)). Ranges in ppm (untransformed) are listed separately for samples taken near the incinerator (< 1 km) and those taken farther away. Elements to be considered for continuous emissions monitoring under SB-488 are bolded. Profile elements included for reference purposes are indicated by †

	Element	log[element] = f (log(distance))			Range in elemental content, ppm			
		xR^2^	Ave N*	Tolerance	Distance < 1 km, *n* = 14		Distance ≥ 1 km, *n* = 22	
					Min	Max	Min	Max
Ref	Al	0.073	6.432	0.251	755.09	3828.25	788.86	2437.81
Emissions profile	**As**	**0.147**	**3.348**	**0.125**	**0.29**	**2.36**	**0.41**	**1.2**
	Ba†	− 0.074	6.432	0.251	25.59	119.67	18.28	118.58
	**Cd**	**0.62**	**12.363**	**0.502**	**0.22**	**6.37**	**0.1**	**0.34**
	Co	0.143	6.432	0.251	0.68	4.75	0.57	2.06
	**Cr**	**0.289**	**9.506**	**0.376**	**1.75**	**13.37**	**1.31**	**4.26**
	Cu	0.342	12.363	0.502	7.89	77.27	5.24	15.63
	Fe	0.146	6.432	0.251	948.42	4931.74	918.33	3427.68
	**Hg**	**0.652**	**9.506**	**0.376**	**0.07**	**0.84**	**0.05**	**0.12**
	**Mn†**	− **0.113**	**29.715**	**2.006**	**52.48**	**613.76**	**82.41**	**286.42**
	Mo	0.371	6.432	0.251	0.34	1.78	0.23	1.55
	**Ni**	**0.34**	**6.432**	**0.251**	**0.98**	**7.18**	**0.85**	**3.45**
	**Pb**	**0.556**	**9.506**	**0.376**	**2.41**	**59.02**	**1.52**	**5.28**
	Sb	0.437	9.506	0.376	0.33	10.84	0.16	14.29
	**Zn**	**0.529**	**9.506**	**0.376**	**44.98**	**540.75**	**26.36**	**69.66**
Rare earth elements	Ce	0.064	6.432	0.251	1.48	6.87	1.68	4.81
	Dy	0.082	6.432	0.251	0.11	0.56	0.1	0.36
	Er	0.12	6.432	0.251	0.06	0.29	0.05	0.19
	Eu	0.342	14.991	0.627	0.04	1.52	0.04	0.12
	Gd	0.154	6.432	0.251	0.15	0.85	0.15	0.48
	La	0.094	6.432	0.251	0.75	3.99	0.87	2.42
	Nd	0.026	6.432	0.251	0.75	3.82	0.82	2.44
	Pr	0.013	6.432	0.251	0.19	0.92	0.21	0.61
	Sm	0.038	6.432	0.251	0.15	0.72	0.15	0.49
	Tm	0.123	6.432	0.251	0.01	0.04	0.01	0.03
	Yb	0.11	6.432	0.251	0.05	0.24	0.04	0.15
Other	Be	0.118	6.432	0.251	0.03	0.13	0.03	0.07
	Cs	0.081	6.432	0.251	0.08	0.37	0.08	0.19
	Ga	0.097	6.432	0.251	0.3	1.52	0.3	0.89
	Rb	0.01	29.715	2.006	3.18	8.91	2.32	8.26
	**Se**	**0.085**	**14.991**	**0.627**	**0.07**	**0.25**	**0.07**	**0.16**
	Sr	− 0.074	9.506	0.376	23.39	66.68	19.91	63.83
	Tl	0.093	6.432	0.251	0.01	0.05	0.01	0.07
	U	0.106	6.432	0.251	0.04	0.23	0.05	0.12
	V	0.102	6.432	0.251	4.45	15.21	3.7	13.71

**Fig. 2:**
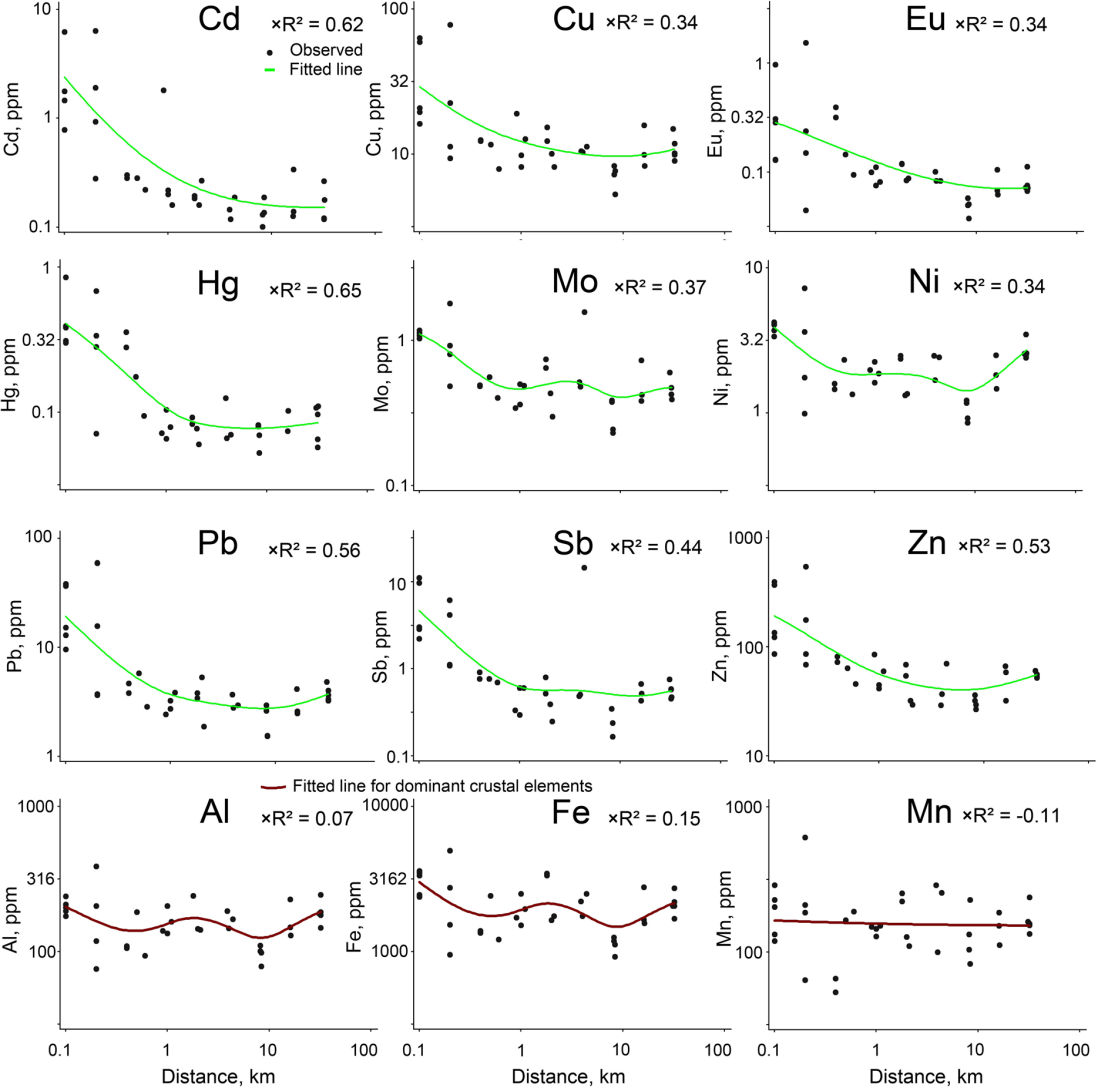
Elemental content (ppm) of the moss *Orthotrichum* in relation to distance (km) from the incinerator. Both elemental concentrations and distances are on log scales. All elements with cross-validated R^2^ (× *R*^2^) > 0.30 are included in the top three rows. The bottom row includes dominant crustal elements (Al, Fe) and Mn for comparison; these were not patterned relative to the incinerator and presumably driven by local patterns of airborne soil or other factors for Mn. The fitted line is based on nonparametric regression with a kernel smoother, the smoothing parameter optimized to maximize × *R*^2^. Further model statistics and data spreads are in Table 1

The spatial scale and shape of NPR models varied by element. The response curves of stronger models (× *R*^2^ > 0.30) tended to have an inflection point near 1 km and an asymptote within 5–10 km (Fig. 2). In contrast, profile elements with weaker models, like As, Co, and Cr, tended to be edaphic elements varying at smaller spatial scales along the transect, with localized peaks within 0.2 km (Online Resource 5). Distributions of the major crustal elements Al and Fe also varied at smaller spatial scales (Fig. 2), although Fe, also a profile element, peaked adjacent to the facility like the edaphic elements.

**Fig. 3:**
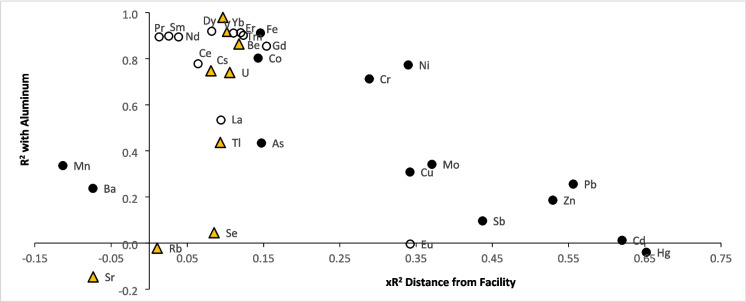
Scatterplot of the strengths of each element’s relationship to aluminum (*R*^2^ from linear regression) versus distance from the incinerator (× *R*^2^ from nonparametric regression). Elements in the emissions profile are indicated by black circles, rare earth elements by open circles, and other trace elements by orange triangles

Accordingly, Al had little spatial correlation to the facility (× *R*^2^ = 0.07) while the model for Fe just made our cut-off for further consideration (× *R*^2^ = 0.15; Table 1). Only two REEs, Eu (× *R*^2^ = 0.34) and Gd (× *R*^2^ = 0.15), had models with equal or stronger fits. Elements that were tightly correlated with Al, like most REEs, also had relatively weak relationships with distance from the incinerator (Fig. 3).

### Correction for geogenic sources

Correcting elemental content for Al before relating to distance from the incinerator had mixed results. Of the eight tested elements, three showed a substantial increase in fit with distance to the incinerator (Cr, Cs, Ni), four showed a small increase in fit with distance (As, Cu, Ce, U), and one showed a decline in fit (Co). Response curves showed individualistic and enigmatic patterns for some elements (Online Resource 6). Only Cr and Cu showed nearly monotonic declines in concentration with distance, the pattern expected if the incinerator was a major large-scale source of the elements. For the two REEs with the strongest correlations (Eu and Gd), correcting for Ce concentration did not increase the strength of those relationships.

### Additional analysis for stakeholders

**Fig. 4:**
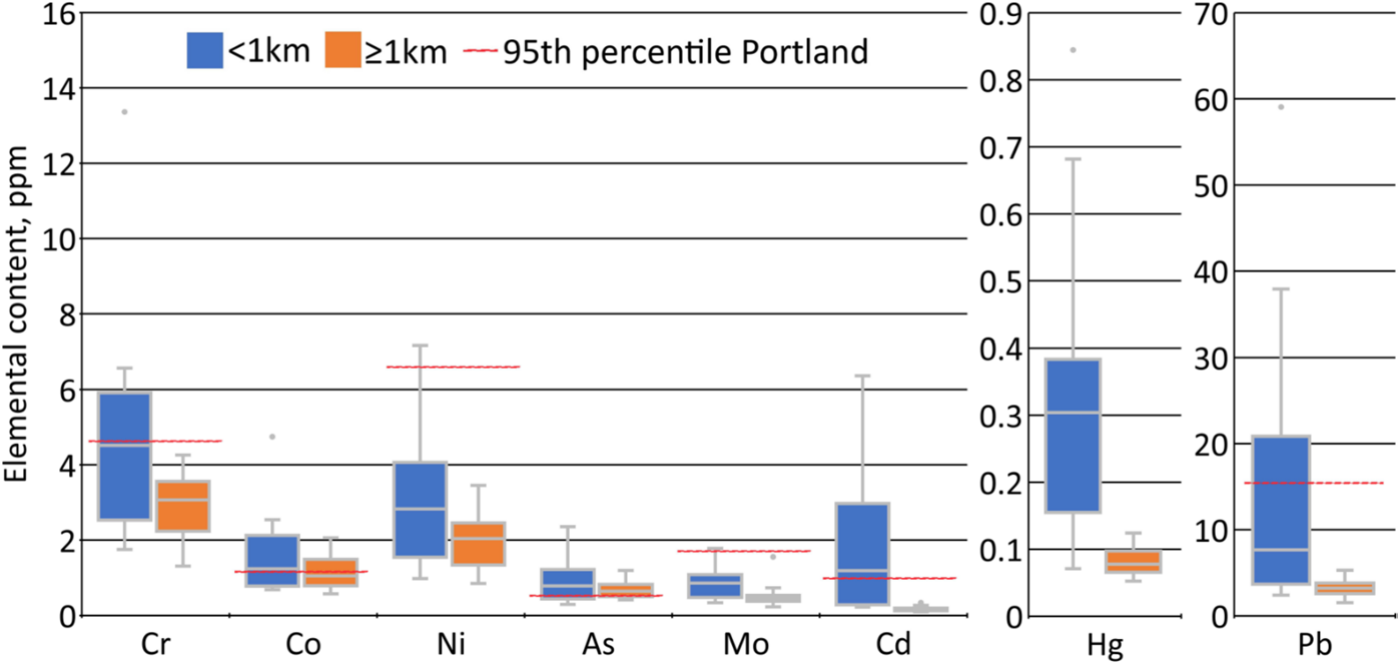
Boxplots for priority elements in *Orthotrichum* spp. for nearer (< 1 km) and farther (≥ 1 km) sites on the transect. Maps of concentrations are provided in Online Resource 5. 95th percentiles from the city-wide Portland survey are provided as reference points for all elements except Hg, which was first measured in the current study

The boxplots showed that all priority elements tended to be higher at sites within 1 km of the facility (Fig. 4). Accordingly, all exceedances of the Portland 95th percentiles occurred within 1 km, except for Co and As. These two elements exceeded their percentiles at most sample sites.

## Discussion

The screening dataset resolved a chemically and spatially complex footprint consistent with expectations for SWI emissions, seeming to confirm the community’s inquiry about air quality effects. Results also supported using *Orthotrichum* spp. as a screening tool for SWI with some caveats, discussed further in the “Study limitations” section. The elemental signatures in moss strongly resembled the emissions profile except for the reference elements Mn and Ba as expected. Otherwise, the strengths and spatial scales of associations between profile elements and the facility varied, with the highest concentrations consistently measured in moss near the facility. A similar pattern was also seen for Ag (Online Resource 5), the one profile element we excluded from statistical analysis because concentrations were below the detection limit at many sites (Online Resource 4). In addition to extensive use in electronics, silver nanoparticles are used in textiles and some medical devices as an antimicrobial (Padhye et al., 2023). To the best of our knowledge, these results provide the first evidence of trace element contamination in the area, serving as an empirical starting point for stakeholders to discuss regulatory and community concerns.

### Spatial scales and emissions pathways

The strong spatial relationships for Cd, Hg, and Pb are consistent with their prevalence in SWI emissions (Linak & Wendt, 1993; National Research Council, 2000). Additionally, the most highly correlated element, Hg, is often used in lichen biomonitoring studies as an atmospheric tracer of SWI emissions (e.g., Contardo et al., 2018; Tretiach et al., 2011). Concentrations of all three elements in *Orthotrichum* spp. declined rapidly with distance from the facility (Fig. 2), as commonly seen in biomonitoring studies around point sources (Fernández et al., 2007; Varela et al., 2014). Similar patterns were also observed in incinerator studies using epiphytic lichens for Hg (Fortuna et al., 2019) and for Cd and Pb in the multi-element study by Loppi et al. (2000). While trace metals may be emitted through either or both pathways (stack or fugitive dust from ash), Hg and Cd are primarily stack-emitted while Fe is mainly from ash (Bolan et al., 2023; Chang et al., 2000). Thus, the similar multi-kilometer distributions we observed for several other elements in the emissions profile, such as Pb, Cu, Sb, and Zn, are suggestive of stack emissions (Fig. 2).

A similar distribution was observed for Eu, an REE widely used for its phosphorescent properties in television and computer screens and low-energy lamps. Europium is also used in plastics as a laser material and in making thin super-conducting alloys (Royal Society of Chemistry, 2024). We found no prior research, biomonitoring or otherwise, associating Eu with incinerator emissions. The closest related studies were prospecting efforts suggesting Eu and other REEs can be economically upcycled from incinerator fly ash (Morf et al., 2013; Wen et al., 2024), although our results indicate that not all REE material ends up trapped in the ash. Potential stack emissions of Eu merit further study and may establish Eu as a useful tracer of SWI emissions. As the economic importance of REEs grows, their prevalence in the waste stream of incinerators will presumably also increase.

### Edaphic elements

The reference element Al was largely unrelated to distance from the incinerator, as expected for crustal elements of geogenic origin (e.g., Loppi et al., 2001). Unsurprisingly, edaphic elements closely tracked Al, including most REEs, indicating the incinerator was not a substantial source compared to soil-based sources (Fig. 3; Urošević et al., 2020). It is possible, however, that the localized associations observed for edaphic elements in the emissions profile (i.e., As, Co, Fe, and Cr) indicate fugitive dust emissions. Those elements are highly abundant in the ash (Bolan et al., 2023) and comparable results were found for Fe and Cr in the similar study by Loppi et al. (2000).

Interestingly, the REE Gd had a similar localized association with the facility. This element is used as a contrast reagent for magnetic resonance imaging (MRI) and thus may reflect medical waste incineration by the facility more specifically than the other elements we tested. Information about Gd as an environmental pollutant focuses on the contamination of aquatic systems by hospital effluents (Ognard et al., 2021), so its potential as an atmospheric tracer has not been explored.

### Correction for geogenic sources

There are several methods for correcting element concentrations for geogenic inputs although none are widely accepted as best (Giráldez et al., 2021). If anything, our exploratory analysis using Al mostly strengthened spatial associations consistent with the incinerator as a source. This included three of the four edaphic elements we tested from the emissions profile (As, Cr, Ni). The exception was Co, where correcting halved the spatial association, suggesting inputs were mainly from natural, soil-based sources. This outcome was not surprising given the strong relationship to Al (Fig. 3). The most striking change was for Cr, where model strength doubled (xR^2^ = 0.29 to 0.59) and described a larger-scale association with the facility than the localized peak observed using raw data (Online Resource 6).

Correcting also improved spatial associations for Cs and marginally for U, the latter model remaining weak (xR^2^ = 0.13). Previous studies have used these elements for biomonitoring nuclear fallout (Anderson et al., 2022; Vosel et al., 2021), although it should be noted that we did not differentiate between the isotopes of Cs, one of which is not radioactive. While these results were not statistically strong, they recommend further study of U and especially Cs as SWI tracers.

### Tool utility and generalizability

The screening dataset collected with the help of community volunteers provided complex, spatially detailed information with clear relevance to community concerns and regulatory matters discussed further in “Stakeholder implications” section. The generic emissions profile we used was sufficient to connect element signatures in moss to SWI, although in more routine applications, any on-site emissions monitoring data, as well as emissions inventories (now required by CAO) would ideally inform the selection of elements to measure. Regardless, measuring a wide array of elements is beneficial for cross-referencing emissions datasets since the exact chemical composition of all materials in an incinerator’s waste stream is difficult to know. Likewise, chemically diverse datasets may help identify novel elements to consider in future biomonitoring studies of SWI, like Eu, Gd, and Cs in this study. Screening data can also cross-check pollution prediction maps produced by computational modeling of on-site emissions data (e.g., Donovan et al., 2016; Gatziolis et al., 2016), although we were unaware of any such maps relevant to the current study.

Given our success with *Orthotrichum* spp., it seems surprising that only one other biomonitoring study captured a multi-element signature clearly associated with an SWI. The varied methods and environmental circumstances of previous work make direct comparisons difficult, and relatively few examined signatures rather than single element tracers, like Hg. Regardless, one notable similarity with Loppi et al. (2000) is that both studies took place in rural settings with apparently few other point sources around to confound results. A challenge in urban and industrial studies is clearly differentiating an SWI-specific signature among a variety of possible trace element sources (e.g., Antisari et al., 2011; Paoli et al., 2015). Continued work with tracer elements and developing source apportionment methods could help in these complicated scenarios. Otherwise, failure to resolve an SWI signature could simply mean emissions were not elevated above ambient conditions, as is the goal (e.g., Protano et al., 2015; Wilcke et al., 1997). According to experts, technological advances made over the last decade have greatly reduced SWI emissions (de Titto & Savino, 2019). As the current study demonstrates, biomonitoring can be a valuable cross-check of these assumptions.

### Stakeholder implications

This section is a starting point for communicating how results may inform policy and community concerns around the SWI in this study. In summary, elemental signatures and spatial associations determined using moss indicated SWI as a contributing source of several trace metals at multiple spatial scales. Highly localized associations could be due to fugitive dust while farther dispersing elements would more likely signify stack emission. However, we cannot rule out contributions from other sources in this observational study. That said, we are not aware of other major point sources for trace elements near the incinerator, including any additional facilities holding Title V permits. The surrounding area is mainly comprised of agricultural and residential land (Online Resource 1).

Determining the origin of trace elements farther away on the transect (> 1 km) is more uncertain but less consequential since moss concentrations were lower for most elements (Fig. 4). Many elements gradually increase at the farthest sites, possibly from closer proximity to the Portland metropolitan area or other source(s) not considered in this study. It is important to note that the axes of all graphs in Fig. 2 were log-transformed, which visually emphasizes nearer sites on the transect. Curves for many of the farther dispersing elements suggested most deposition occurs within 5 to 10 km of the incinerator. In several cases, the steepest part of the curves, and thus the most acute deposition levels, occurred within 1 km of the facility.

As a screening tool, we interpret the elemental content of moss mainly in relative, spatial terms (i.e., comparing where concentrations are relatively high vs. low) to help guide the need for further investigation. As initial context, however, we included the 95th percentiles from the Portland moss study for comparison (Fig. 4; Gatziolis et al., 2016). In some cases, hotspots with element concentrations in moss at or above these percentiles were found to have air concentrations exceeding state health benchmarks when air monitors were moved to those locations (Donovan et al., 2016; Jovan et al., 2022). Among the elements with values exceeding these thresholds in the current study, Cd, Pb, and Cr exceedances were limited to a subset of the nearer sites. Results for the strongly edaphic (i.e., soil-influenced) elements As and Co appear more severe but should be interpreted conservatively because soil and wind-blown particles naturally accumulate more during hot and dry conditions. Moss sampling for the current study occurred during a major summer heat wave whereas the Portland dataset was collected during the extended wet winter season. Additionally, geogenic As is naturally high in the study region due to volcanic soils (Hinkle & Polette, 1999) and while less is known about Co in soils, its close relationship to Al in this study suggests much of it is geogenic, at least in the dry season (Fig. 3). In contrast, these seasonal differences are relatively unimportant for non-edaphic elements that mosses retain over long time periods, like Cd and Pb (Boquete et al., 2015; Jovan et al., unpublished data for *Orthotrichum* spp.).

Results may inform three regulatory matters, including how the new continuous emissions monitoring requirements set by SB-488 are implemented. All metals listed in SB-488 were moderately to strongly spatially correlated with the facility (As, Cd, Cr, Hg, Ni, Pb, Zn) besides Mn (not reliable in moss) and Se, which was low compared to previous moss screening studies. Several metals not listed in SB-488 were also spatially correlated, including potential stack emissions of the REE Eu and localized fugitive dust emissions of Gd, the latter possibly specific to medical waste incineration by the facility. We also had weak results suggesting the incinerator as a possible source of U and Cs. Additional uses for these results include helping cross-reference the facility’s emissions inventory required by CAO program and informing the facility’s Title V permit for emitting HAPs, which is up for renewal.

### Study limitations

This study has several limitations. As mentioned previously, concentrations of air contaminants in moss or lichens do not reliably predict measurements of air quality, and therefore do not track the human health and regulatory thresholds decision-makers use to evaluate whether pollution levels are problematic. Screening results are intended to guide the next phase of investigation. However, potential stress from seeing contaminant levels in biomonitors without immediately understanding their personal or regulatory importance, if any, is a major disadvantage for applying this tool in “real world” situations. To help moderate concerns in the current study, we included a provisional analysis for stakeholders showing that only a minority of moss samples had relatively high contaminant levels compared with prior screening data, and these mainly came from sites close to the incinerator.

A second limitation concerns the long, uncertain retention times of contaminants in native biomonitors. Long retention times can be beneficial, as in the current study, since SWI emissions vary widely over time (National Research Council, 2000). On the other hand, the utility of native material for monitoring temporal changes in contaminant levels is limited unless broadly defined, long-term differences are of interest or potentially in special cases where it is possible to identify and harvest only newer growth on the biomonitors (e.g., Loppi et al., 2000 or studies using *Hylocomium splendens*). Studies evaluating temporal change often use transplanted material instead because the timeframe represented can be precisely controlled. One option for transplants is using the European cloned *Sphagnum* “Mosspheres,” which standardize some of the physical characteristics known to affect trace metal bioaccumulation (Vázquez-Arias et al., 2024).

Finally, our target species group, *Orthotrichum* s.l., does not grow everywhere, although it is broadly distributed in the Northern Hemisphere in temperate climates. If *Orthotrichum* is not an abundant epiphytic moss available for a screening study, substitution of another species that is well distributed and regionally abundant would be required. A variety of lichen and moss species perform well for trace metals biomonitoring (Abas 2021; Chaudhuri & Roy, 2023), and depending on study goals, transplants are always an option.

## Conclusions

This study provides new evidence supporting the use of native epiphytic moss, specifically *Orthotrichum* spp., as a biomonitoring tool for assessing trace elements around SWIs. By analyzing a comprehensive suite of elements, including REEs, we demonstrated that moss can capture the complex, spatially resolved pollution footprint expected from incinerator emissions. Common stack-associated elements, like Cd, Hg, and Pb, had the strongest geographic associations in this study while localized peaks of edaphic elements, like As and Cr, emphasizes the need for further research into biomonitoring fugitive dust emissions from SWI. The study also highlights novel atmospheric tracers for SWI, including Cs, Eu, and Gd, with the latter potentially specific to the facility’s incineration of some medical waste. Additional investigation of SWI emissions of these elements, and REEs more generally, is recommended.

Overall, results underscore the utility of native moss as a cost-effective screening tool, capable of identifying spatial pollution patterns that can guide regulatory and community actions. Arguably, lack of communication with potential users is a critical roadblock to applying results to real world issues, and including such guidance in journal publications is one strategy to help overcome this. In this case, we linked findings to specific issues concerning the studied facility in subsections aimed at stakeholders.

## Supplementary Information

Below is the link to the electronic supplementary material.

Supplementary file1 (XLSX 69 KB)

Supplementary file2 (DOCX 4262 KB)

## Data Availability

Study data are provided in the supplementary information file Supplementary file 1.
