# Correction to: Putting biomonitors to work: native moss as a screening tool for solid waste incineration

**DOI:** 10.1007/s10661-025-14144-w

**Published:** 2025-05-27

**Authors:** Sarah Jovan, Eleonore Jacobson, Jason M. Unrine, Nasser Jalili-Jahani, Bruce McCune

**Affiliations:** 1 U.S. Forest Service, Pacific Northwest Research Station, Portland, OR USA; 2 Department of Botany and Plant Pathology, Oregon State University, Corvallis, OR 97331 USA; 3 Department of Plant and Soil Sciences, University of Kentucky, Lexington, KY 40546 USA; 4 University of Kentucky, Kentucky Water Research Institute, Lexington, KY 40546 USA

**Correction to: Environ Monit Assess (2024) 196:1177**

10.1007/s10661-024-13354-y

In the Acknowledgements section of the article, some names are misspelled.

The name "Carrol" should be corrected to "Carroll" and the name "Ohman" should be corrected to "Ohmann". The corrected Acknowledgements section is shown below.

**Acknowledgements** We would like to thank Janet Ohmann, Zane Walker, and Faye Yoshihara for their invaluable assistance in sample collection. We would also like to thank Zane Walker, Claire Whittaker, and Faye Yoshihara for their help processing moss samples, and Lisa Arkin and Carroll Johnston for discussion relevant to the study. We also appreciate comments from 2 anonymous reviewers that improved the clarity of the manuscript.

