# Supplementary material for: Putting biomonitors to work: native moss as a screening tool for solid waste incineration
Source: Environ Monit Assess. 2024 Nov 7;196(12):1177. doi: 10.1007/s10661-024-13354-y (PMC11543719; doi:10.1007/s10661-024-13354-y)
Supplement: Supplementary file 2 — Supplementary file2 (DOCX 4262 KB) [file 10661_2024_13354_MOESM2_ESM.docx]

**Supplementary materials**

Putting biomonitors to work: tracing emissions from solid waste incineration with native moss

Environmental Monitoring and Assessment

Sarah Jovan, U.S. Forest Service, Pacific Northwest Research Station, Portland, Oregon, US; sarah.jovan@usda.gov; ORCID 0000-0001-7860-4005, Eleonore Jacobson, Jason M. Unrine, Nasser Jalili-Jahani, Bruce McCune

**Online Resource 1**

**Fig. 1:** Maps of the study area showing the sampling transect (top) and detail of sites nearest the facility (bottom). The basemap is satellite imagery from 10/14/2022

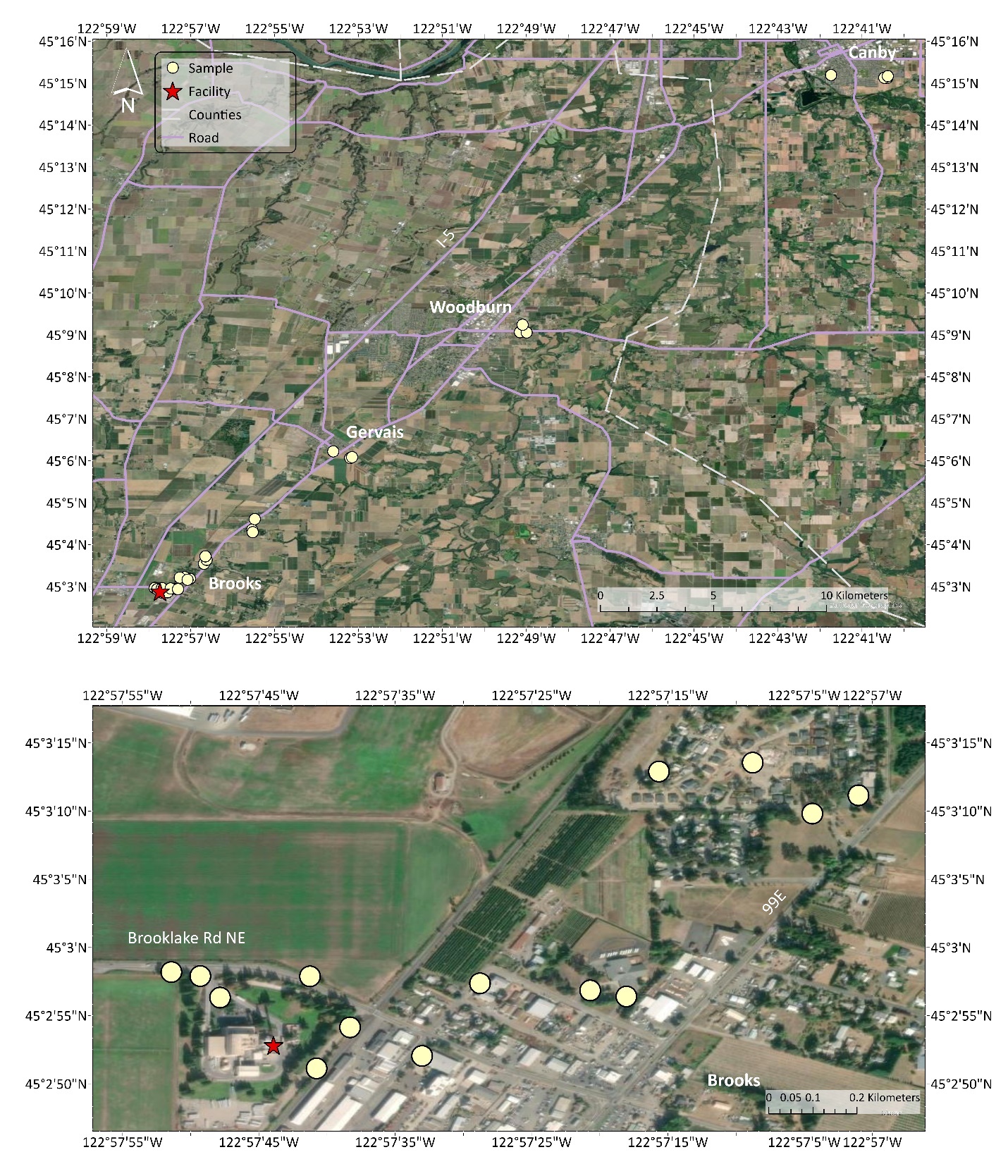


**Online Resource 1 (continued)**

**Fig. 2:** 10-year wind roses for the study area (2012-2022), including: A) Aurora State AP and B) Salem AP (McNary Field). The study transect was oriented to the NE to align with the dominant W to SW winds.

**
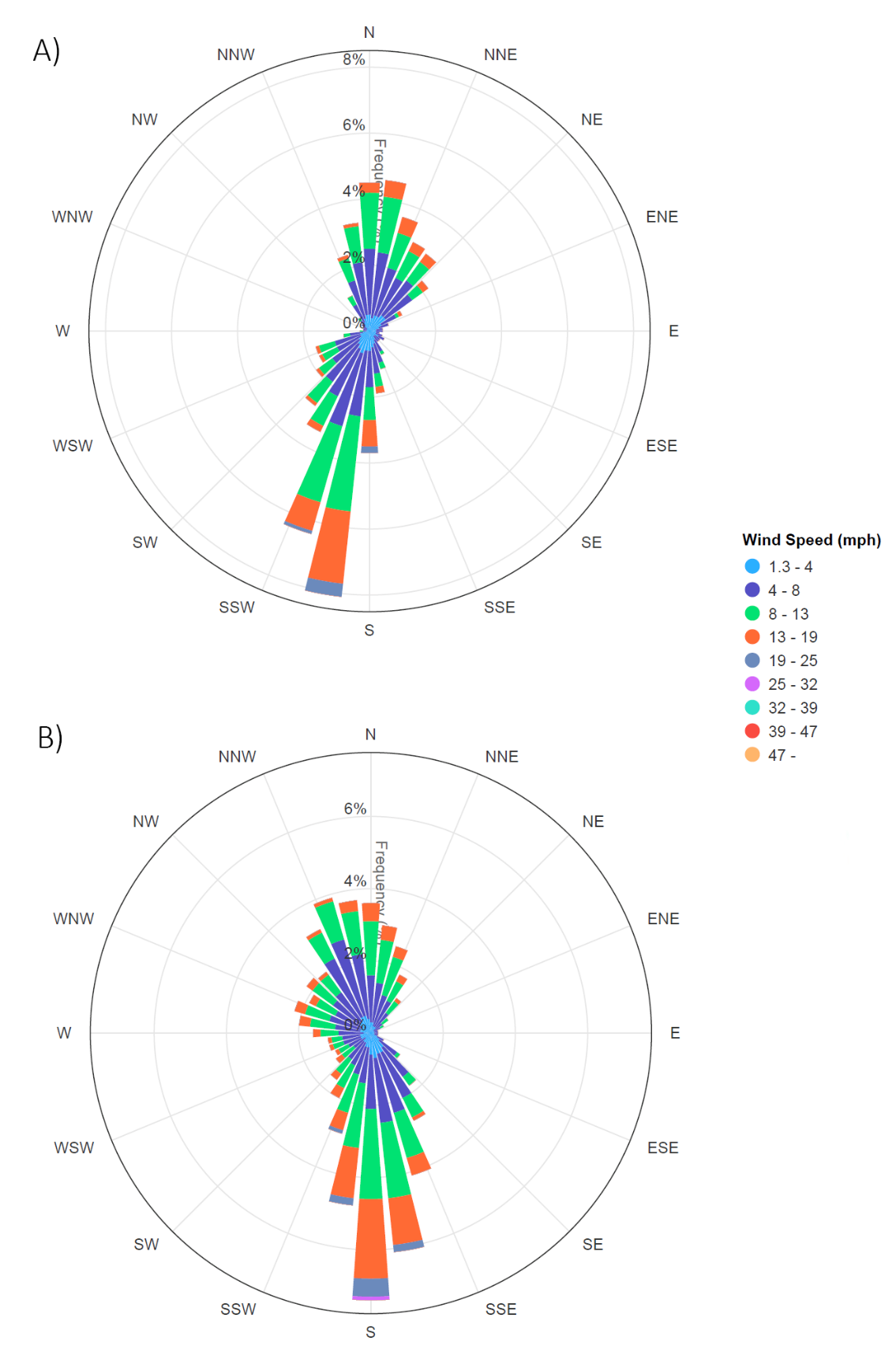
**

**Online Resource 2 –** Synopsis of the sample processing protocol

To minimize contamination, a laminar flow hood and surface cleaned with 70% ethanol was used for preparing the samples before sending to the lab conducting the trace metals analysis. After the Kapak bags used to collect the samples were opened, moss contents were distributed onto a polyurethane petri dish. At least two polyurethane petri dishes were used during the process of cleaning a sample; one to two dishes were used to distribute the sample, and the third was used to collect prepared moss. New nitrile gloves and polyurethane petri dishes were used for each sample. Samples were prepped by cutting moss stems and removing discolored tissue, foreign debris, insects, and dead tissue using ceramic scissors. Under a fume hood, the ceramic scissors were rinsed between samples with 10% citranox, then deionized water, then a 10% nitric acid (trace-metal grade) solution, deionized water again, and then dried with a Kim wipe. The nitric acid and citranox solutions were prepared using deionized water. Between sample preparations, the surface under the laminar flow hood was cleaned again with 70% ethanol. Prepared moss was transferred to a fresh Kapak bag, sealed with new painter’s tape, and the weight was recorded. Samples were stored at 4^o^C then mailed to University of Kentucky for ICP-MS analysis.

**Online Resource 3 –** Summary of elemental recovery data from National Institute of Standards and Technology Standard Reference Materials.

|  | **SRM 1515 Apple leaves** | | |  |  |  | **SRM 1537a Tomato leaves** | |  |  |  |
| --- | --- | --- | --- | --- | --- | --- | --- | --- | --- | --- | --- |
| **Element** | **Certified or reference value (ug/g)** | **Mean recovery (n = 8)** | **Percent Recovery** | **Standard deviation** | **Coefficient of variation** |  | **Certified or reference value (ug/g)** | **mean recovery (n = 5)** | **Percent Recovery** | **Standard deviation** | **Coefficient of variation** |
|  |  |  |  |  |  |  |  |  |  |  |  |
| **B** | 27.6 | 29.35 | 106% | 4.34 | 14.8% |  | 33.13 | 53.25 | 161% | 1.91 | 3.6% |
| **Al** | 284.5 | 123.29 | 43% | 15.95 | 12.9% |  | 598.4 | 309.27 | 52% | 24.39 | 7.9% |
| **V** | 0.254 | 0.16 | 63% | 0.01 | 8.9% |  | 0.835 | 0.58 | 69% | 0.04 | 6.2% |
| **Cr** | 0.3 | 0.36 | 121% | 0.20 | 56.0% |  | 1.988 | 2.20 | 111% | 0.14 | 6.3% |
| **Mn** | 54.1 | 53.41 | 99% | 1.72 | 3.2% |  | 246.3 | 247.86 | 101% | 1.25 | 0.5% |
| **Fe** | 82.7 | 59.69 | 72% | 3.03 | 5.1% |  | 367.5 | 281.86 | 77% | 5.31 | 1.9% |
| **Co** | 0.09 | 0.09 | 103% | 0.01 | 6.8% |  | 0.5773 | 0.59 | 102% | 0.01 | 0.9% |
| **Ni** | 0.936 | 0.98 | 105% | 0.21 | 20.8% |  | 1.582 | 1.53 | 97% | 0.04 | 2.6% |
| **Cu** | 5.69 | 6.15 | 108% | 0.27 | 4.4% |  | 4.7 | 4.70 | 100% | 0.04 | 0.9% |
| **Zn** | 12.45 | 12.42 | 100% | 0.41 | 3.3% |  | 30.94 | 30.02 | 97% | 0.13 | 0.4% |
| **As** | NA |  |  |  |  |  | 0.1126 | 0.07 | 65% | 0.01 | 8.5% |
| **Se** | NA |  |  |  |  |  | 0.0543 | 0.06 | 120% | 0.01 | 17.5% |
| **Rb** | 10.2 | 9.68 | 95% | 0.26 | 2.7% |  | 14.83 | 14.34 | 97% | 0.09 | 0.6% |
| **Sr** | 25.1 | 25.10 | 100% | 0.66 | 2.6% |  | 85 | 84.47 | 99% | 0.32 | 0.4% |
| **Mo** | 0.095 | 0.09 | 92% | 0.01 | 10.0% |  | 0.46 | 0.36 | 78% | 0.01 | 1.4% |
| **Ag** | NA |  |  |  |  |  | 0.017 | BDL |  |  |  |
| **Cd** | 0.0132 | 0.02 | 163% | 0.00 | 11.1% |  | 1.517 | 1.52 | 100% | 0.01 | 0.8% |
| **Sb** | 0.013 | 0.01 | 92% | 0.00 | 9.2% |  | 0.0619 | 0.04 | 67% | 0.00 | 6.8% |
| **Ba** | 48.8 | 48.34 | 99% | 1.67 | 3.5% |  | 63 | 59.16 | 94% | 0.14 | 0.2% |
| **La** | 20 | 20.46 | 102% | 0.66 | 3.2% |  | 2.3 | 2.26 | 98% | 0.01 | 0.3% |
| **Ce** | 3 | 3.25 | 108% | 0.08 | 2.4% |  | 2 | 1.68 | 84% | 0.04 | 2.3% |
| **Nd** | 17 | 16.86 | 99% | 0.68 | 4.0% |  | NA |  |  |  |  |
| **Sm** | 3 | 2.96 | 99% | 0.11 | 3.9% |  | 0.19 | 0.19 | 101% | 0.00 | 1.3% |
| **Eu** | 0.2 | 0.28 | 142% | 0.01 | 2.6% |  |  | 0.05 |  | 0.00 | 1.1% |
| **Gd** | 3 | 3.28 | 109% | 0.09 | 2.6% |  | 0.17 | 0.20 | 117% | 0.00 | 0.6% |
| **Hg** | 0.0432 | 0.08 | 182% | 0.01 | 7.9% |  | 0.03 | 0.03 | 90% | 0.03 | 96.4% |
| **Pb** | 0.47 | 0.44 | 94% | 0.02 | 4.3% |  | NA |  |  |  |  |
| **Th** | 0.03 | BDL |  |  |  |  | 0.12 |  |  |  |  |
| **U** | 0.01 | 0.01 | 188% | 0.00 | 10% |  | 0.035 | 0.01 | 239% | 0.00 | 16% |

**Online Resource 3 (continued) –**Recoveries were acceptable across a broad range of elements for both SRMs and generally within 1-25% of certified or reference values. The exceptions were for B in tomato leaves (161%), Al in both SRMs, V in both SRMs (43% and 52% in apple leaves and tomato leaves), V (63 and 69%), Fe (72% in tomato leaves), As (65% in tomato leaves), Cd (163% in apple leaves), Sb (67% in tomato leaves), Hg (182% in apple leaves) and U (188% and 239% in apple leaves and tomato leaves).

Lower recovery of V and Fe is attributable to the omission of HCl from the digestion which was chosen to avoid chloride interferences for several elements (Unrine et al. 2019). Higher recoveries of Cd and Hg in apple leaves and U in both SRMs are attributable to the certified concentrations being very close to the method detection limits. Method detection limits were calculated separately for each batch. Mean method detection limits ranged from 0.001 ug/g for several elements to 21.01 ug/g for Al. Mean spike recovery averaged 105.7% across all elements and ranged from 99.6% for V to 124.1% for Ag. Mean relative percent difference between dilutions averaged 7.2% ranging from 3.6% for Cr to 17% for Mo. Subsample variation as measured by relative percent difference between digestion duplicates averaged 14.8 % across all elements ranging from 4.9% in Rb to 80% in Be.

**References**

Unrine, J. M., Slone, S. A., Sanderson, W., Johnson, N., Durbin, E. B., & others. (2019). A case-control study of trace-element status and lung cancer in Appalachian Kentucky. *PLOS ONE, 14*(2), e0212340. https://doi.org/10.1371/journal.pone.0212340

**Online Resource 4 –** see Excel file OnlineResource4_rawdata.xlsx

**Online Resource 5 –** Arsenic (As) measured in moss. Concentrations (ppm) are grouped using Jenk’s natural breaks to help visualize the raw data without log transformation

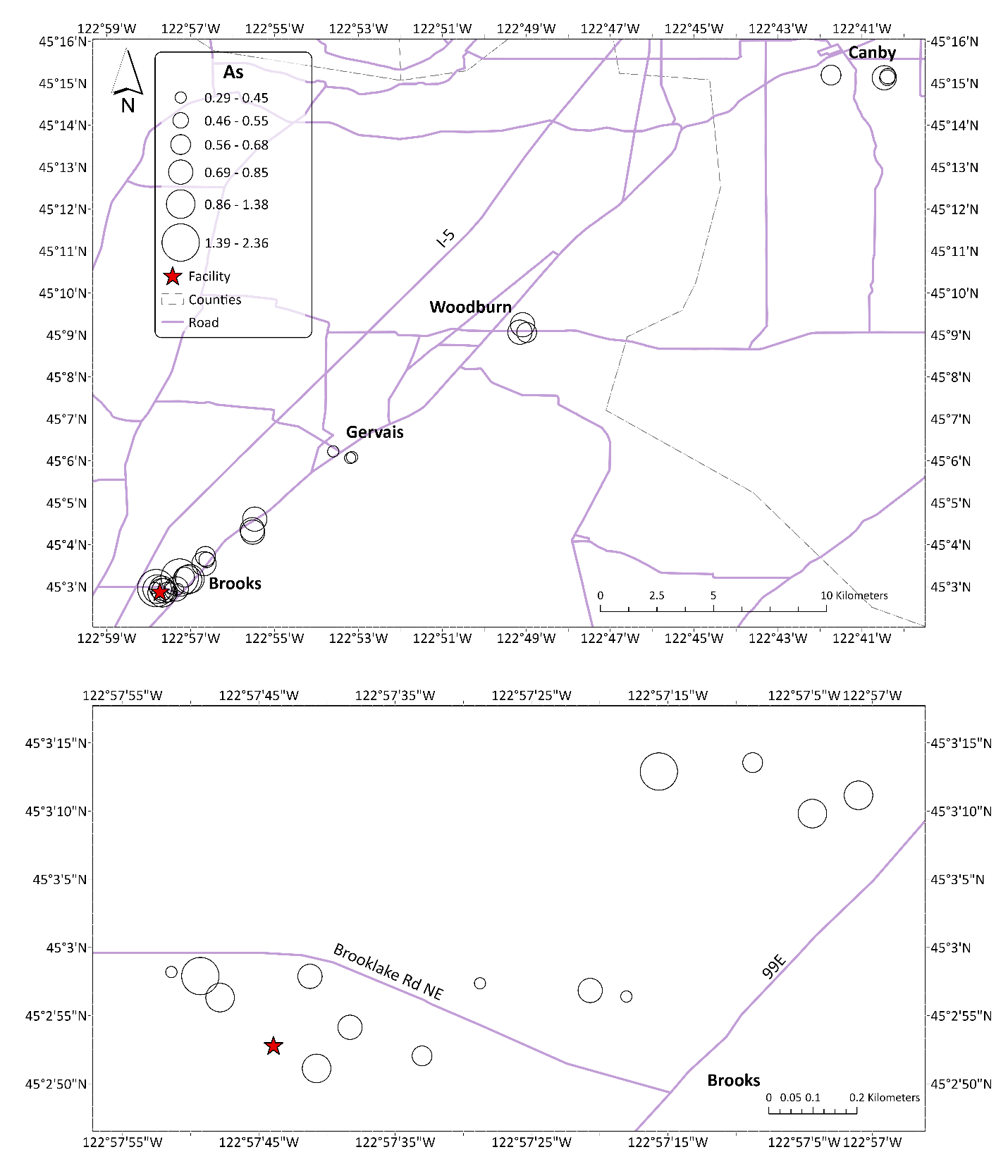


**Online Resource 5 (continued) –** Cadmium (Cd) measured in moss. Concentrations (ppm) are grouped using Jenk’s natural breaks to help visualize the raw data without log transformation

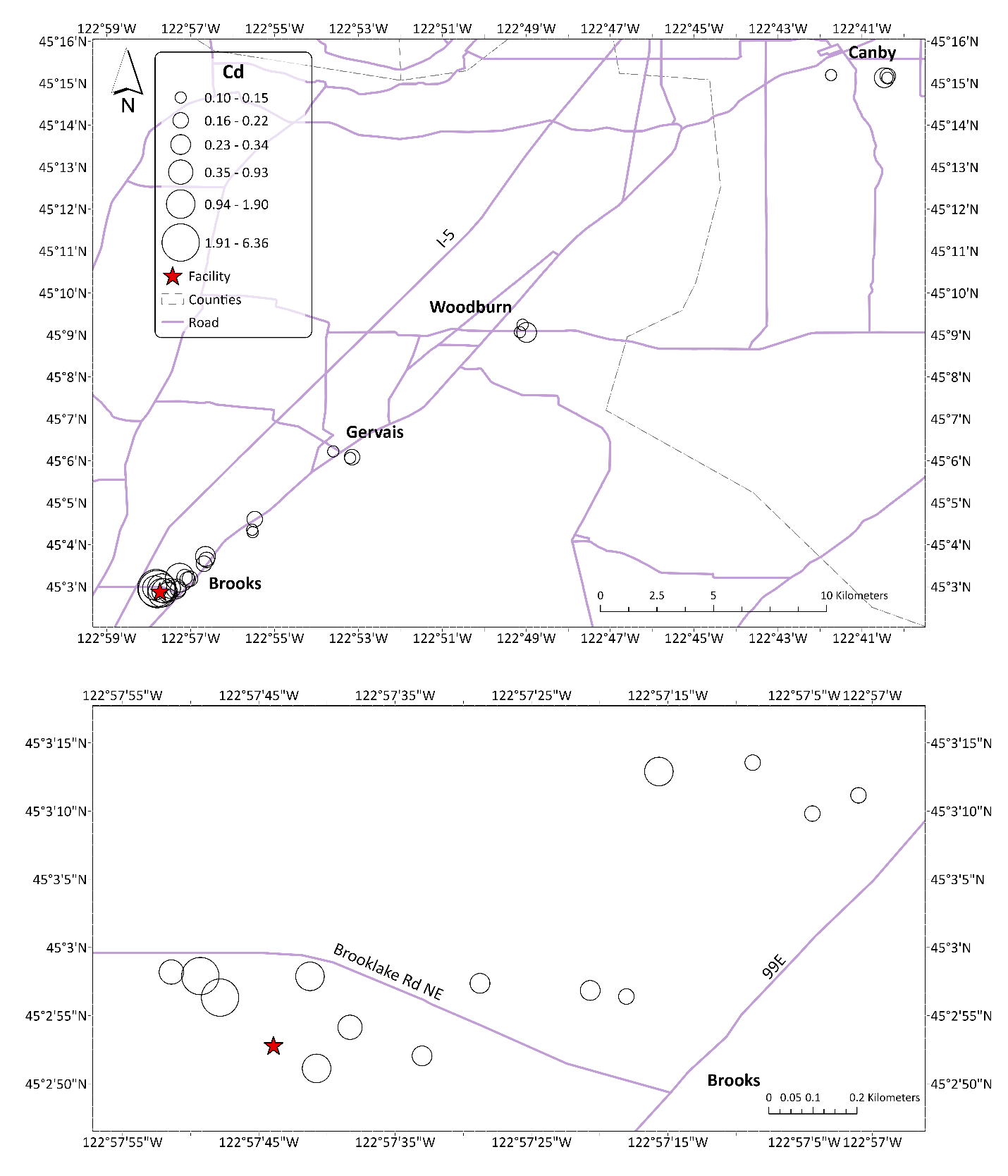


**Online Resource 5 (continued)–** Cobalt (Co) measured in moss. Concentrations (ppm) are grouped using Jenk’s natural breaks to help visualize the raw data without log transformation

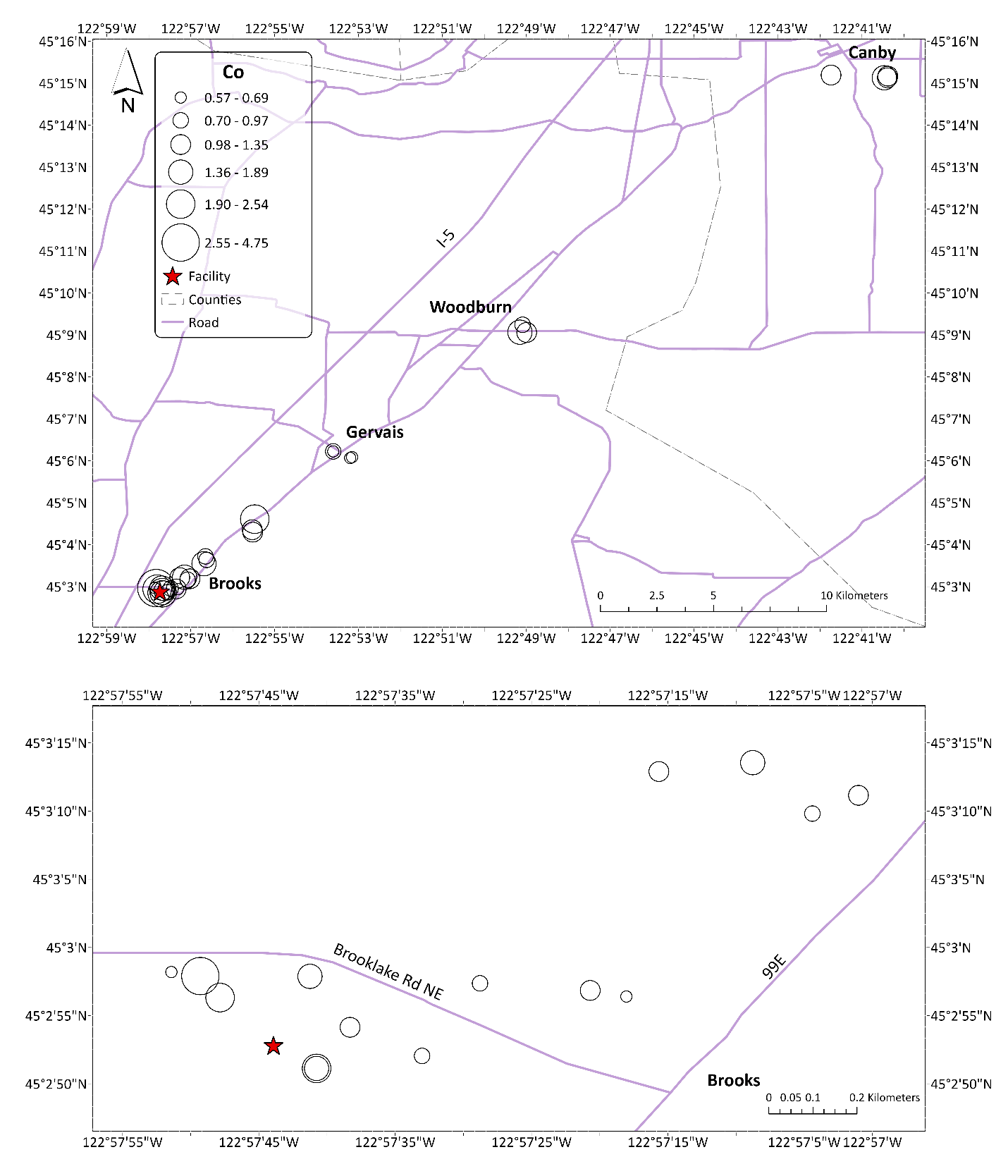


**Online Resource 5 (continued)–** Chromium (Cr) measured in moss. Concentrations (ppm) are grouped using Jenk’s natural breaks to help visualize the raw data without log transformation

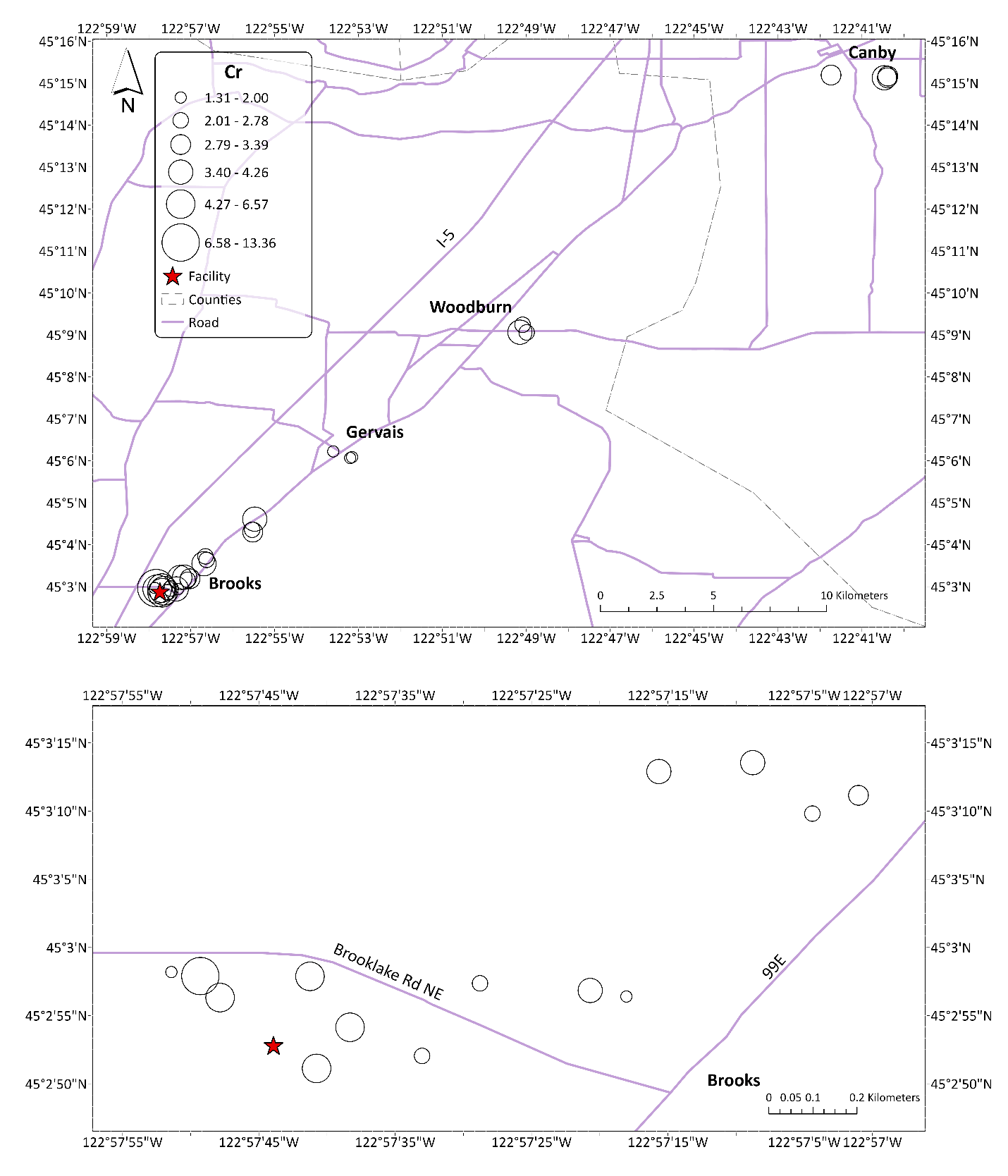


**Online Resource 5 (continued)–** Mercury (Hg) measured in moss. Concentrations (ppm) are grouped using Jenk’s natural breaks to help visualize the raw data without log transformation

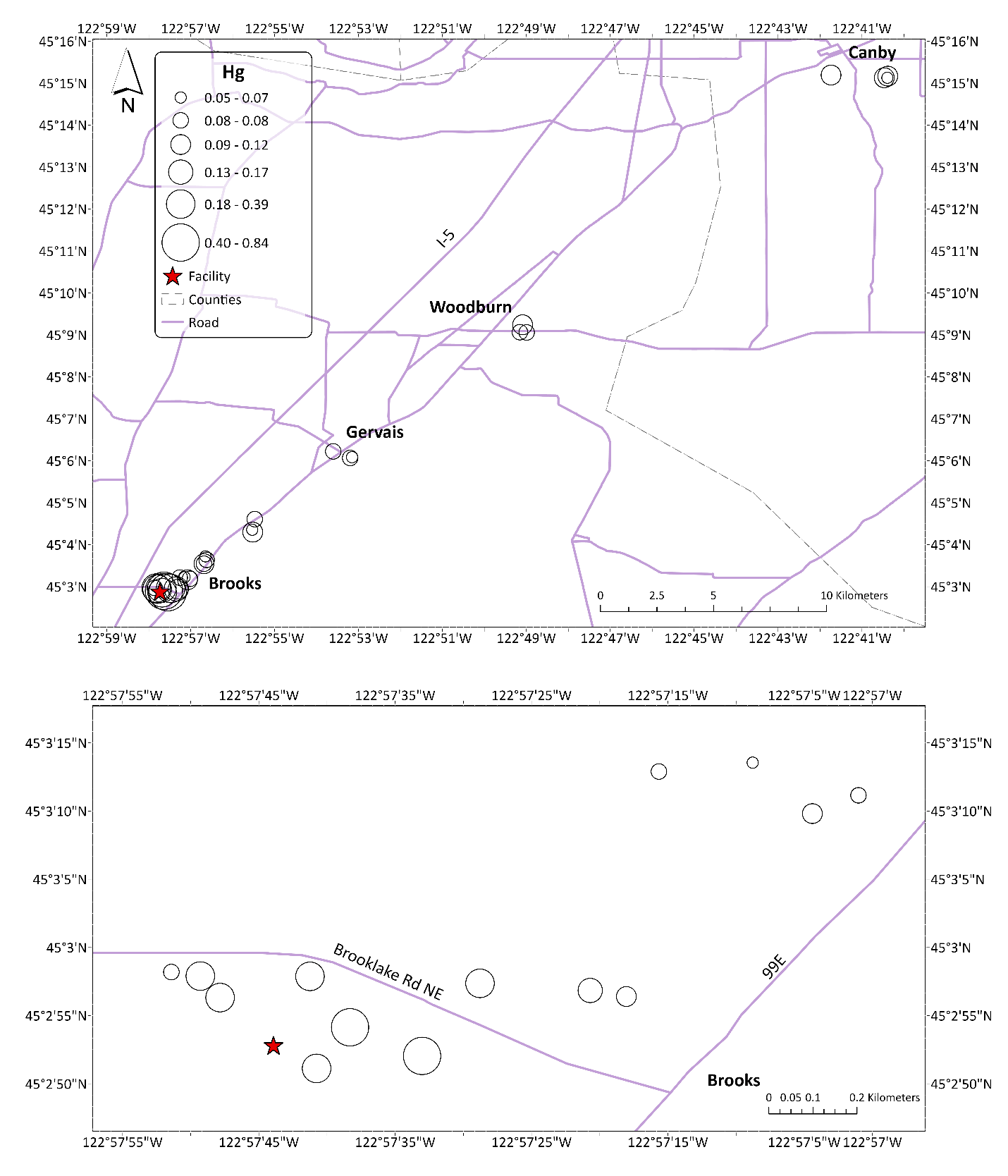


**Online Resource 5 (continued) –**  Molybdenum (Mo) measured in moss. Concentrations (ppm) are grouped using Jenk’s natural breaks to help visualize the raw data without log transformation

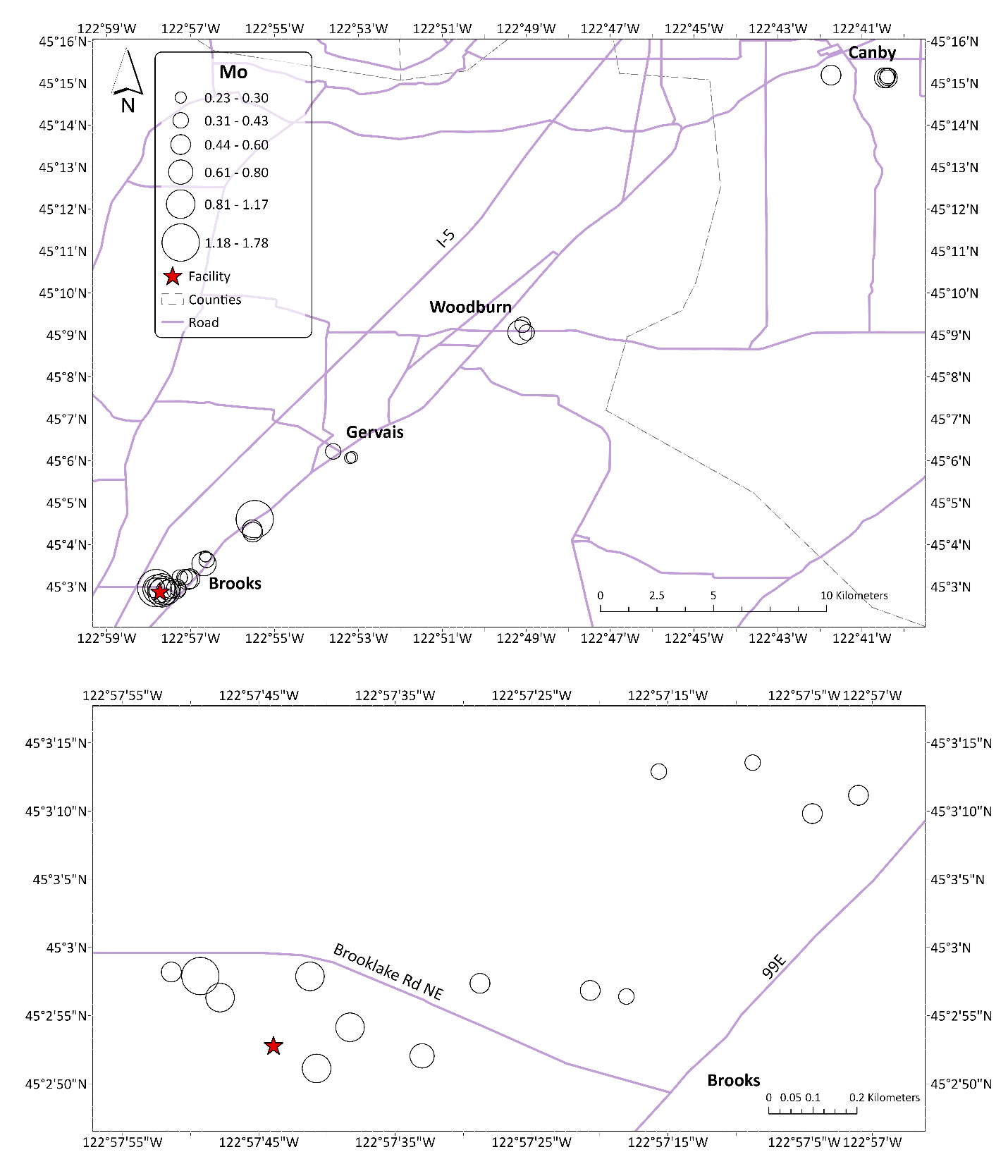


**Online Resource 5 (continued)–** Nickel (Ni) measured in moss. Concentrations (ppm) are grouped using Jenk’s natural breaks to help visualize the raw data without log transformation

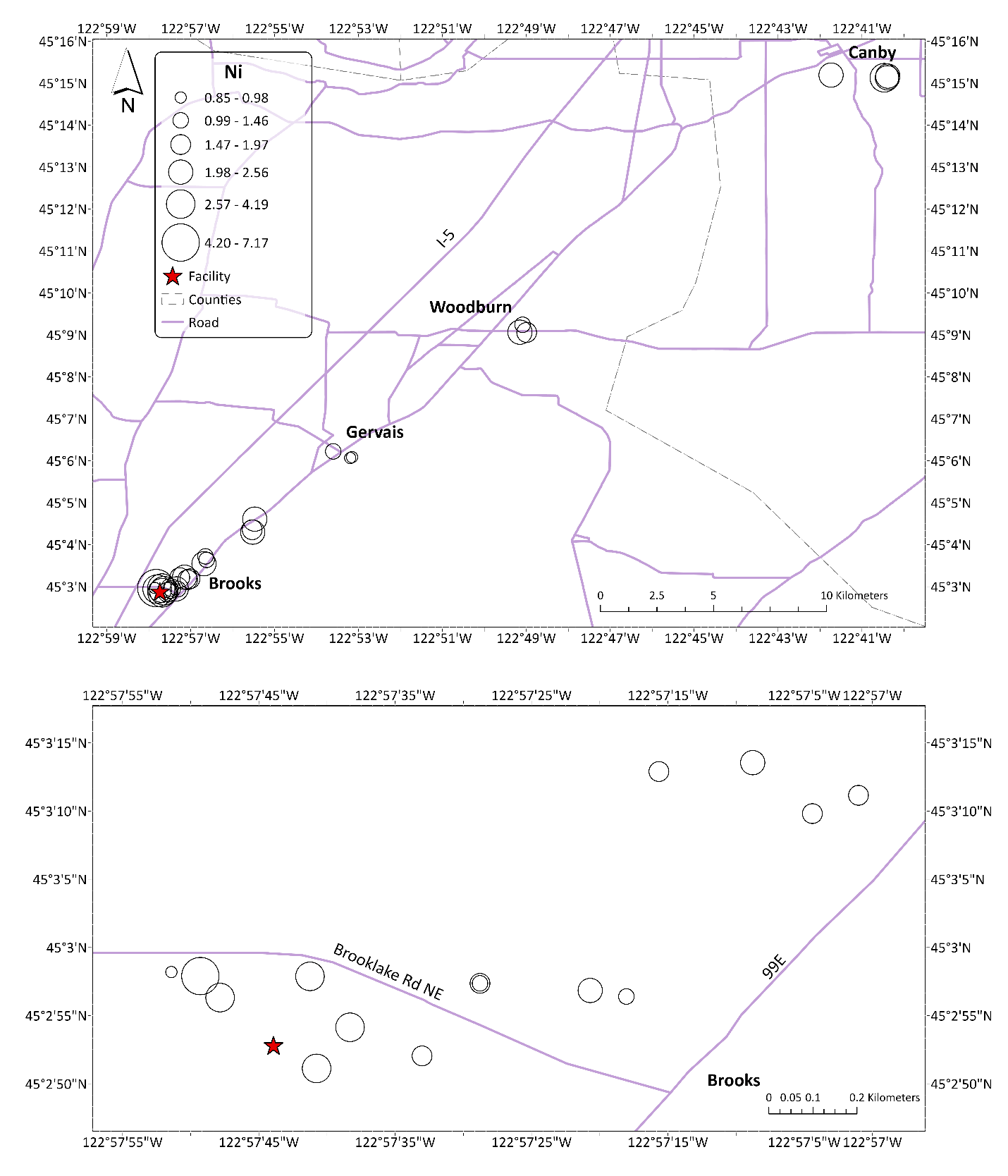


**Online Resource 5 (continued) –**  Lead (Pb) measured in moss. Concentrations (ppm) are grouped using Jenk’s natural breaks to help visualize the raw data without log transformation

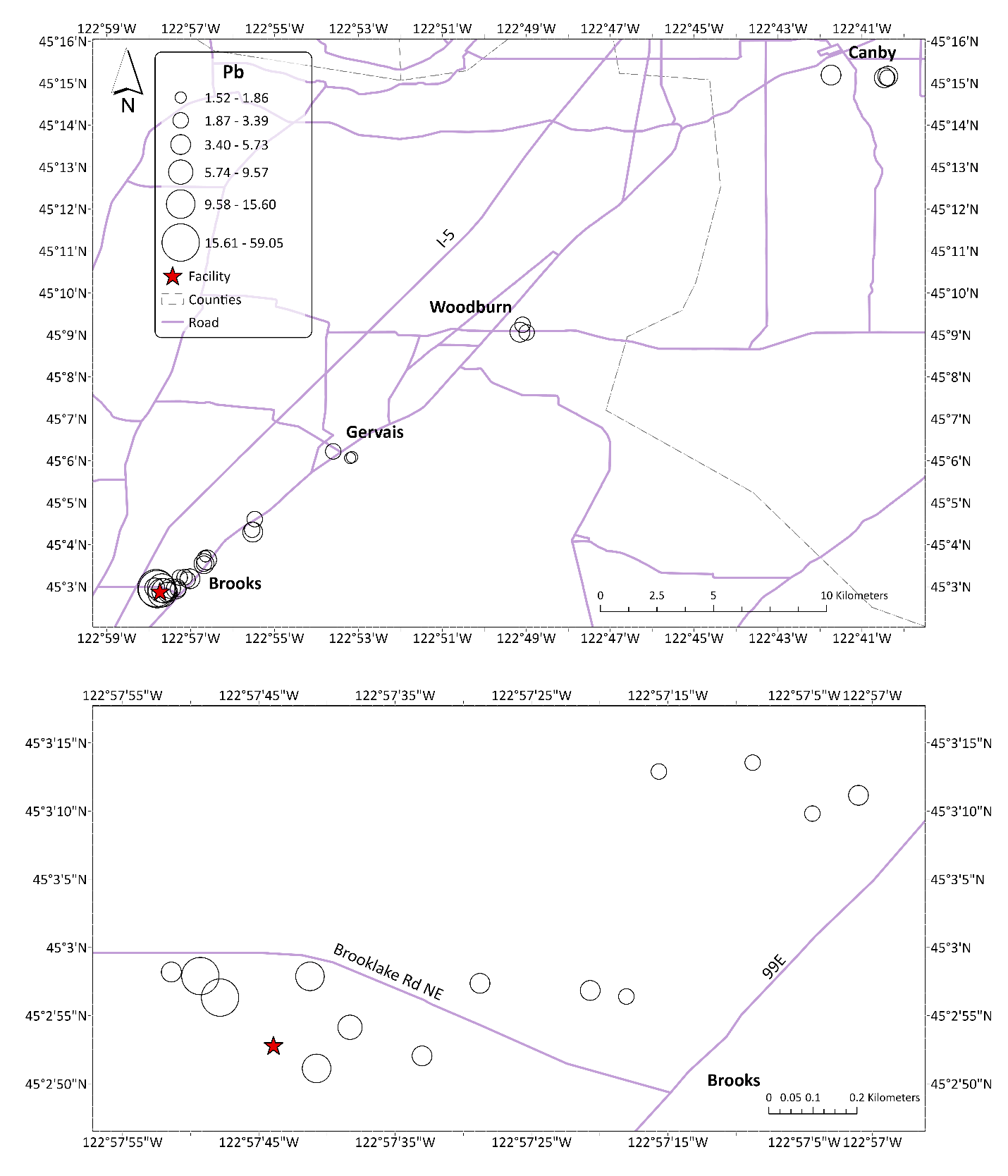


**Online Resource 5 (continued) –**  Silver (Ag) measured in moss. Concentrations (ppm) are grouped using Jenk’s natural breaks to help visualize the raw data without log transformation. Values below the detection limit are denoted as BDL


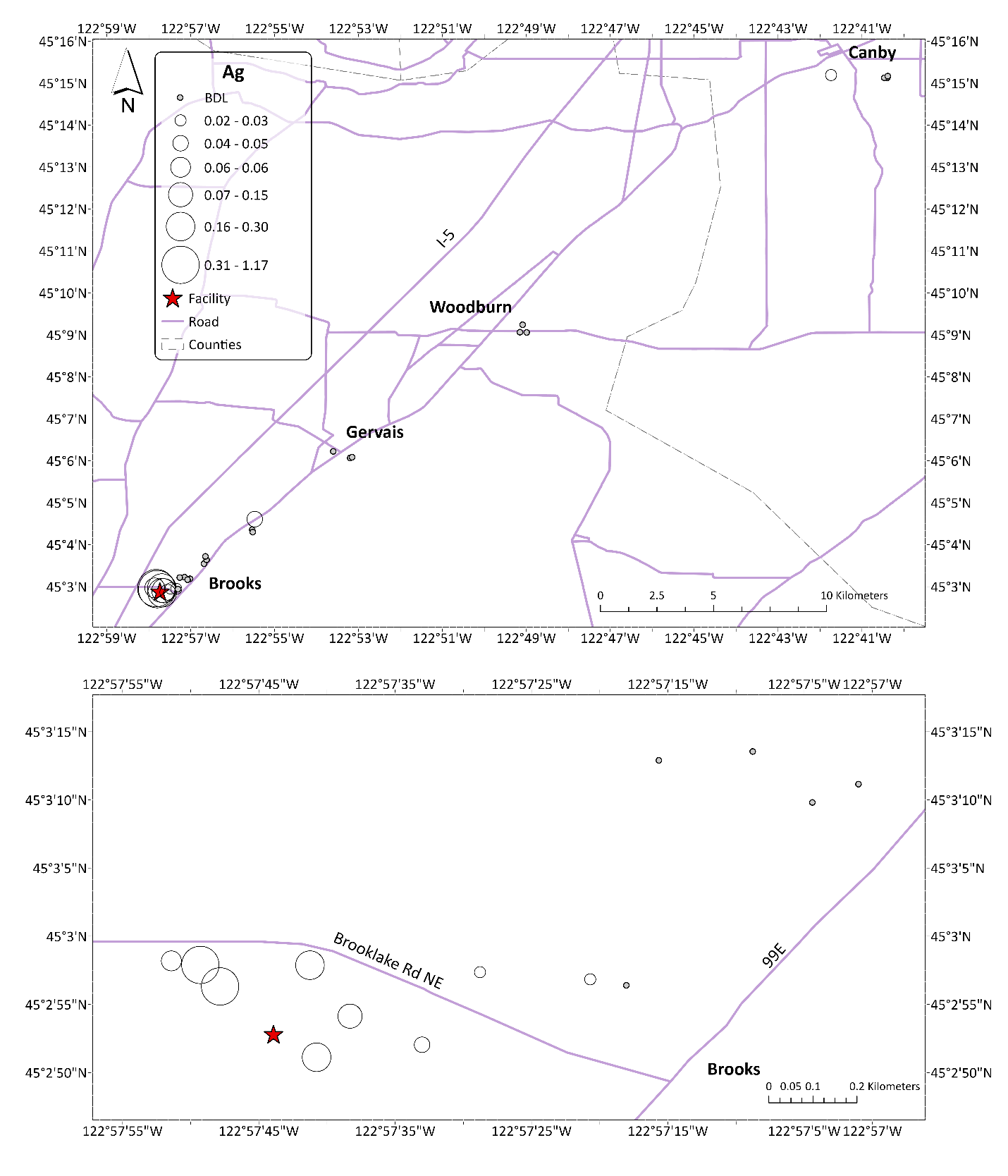


**Online Resource 6 –** Fitted curves and scatterplots for element showing substantially stronger fits to distance from the incinerator after correction for aluminum content. Model fits for all elements are provided in a table on the following page.

|  |  |
| --- | --- |
|  |  |
|  |  |

**Online Resource 6 (continued) –** Fit of elemental content expressed as cross-validated ×*R*^2^ (bold face > 0.30) from nonparametric regressions in relation to: **A**. f(logD), distance to incinerator; **B.** f(Al), aluminum content; **C**. f(logD|Al), distance to incinerator corrected for aluminum content. All variables were log-transformed. Orange highlighted elements met our criteria for performing the adjustment (see Materials and methods). Scatterplots and fitted curves for column C results are shown in a figure on the preceding page.

| **Response** | **A** | **B** | **C** |  |
| --- | --- | --- | --- | --- |
| **Variable, Y** | **Y=f(logD)** | **Y=f(Al)** | **Y=f(logD\|Al)** |  |
| **AsLog** | 0.147175 | **0.434** | 0.225 |  |
| BaLog | -0.07384 | 0.237 |  |  |
| **BeLog** | 0.117751 | **0.864** |  |  |
| CdLog | **0.620161** | 0.012 |  |  |
| **CeLog** | 0.064014 | **0.778** | 0.236 |  |
| **CoLog** | 0.142843 | **0.802** | 0.080 |  |
| **CrLog** | 0.288631 | **0.712** | **0.590** |  |
| **CsLog** | 0.080971 | **0.747** | **0.350** |  |
| **CuLog** | **0.341969** | **0.308** | **0.360** |  |
| **DyLog** | 0.081952 | **0.919** |  |  |
| **ErLog** | 0.120042 | **0.912** |  |  |
| EuLog | **0.342326** | -0.004 |  |  |
| **FeLog** | 0.145867 | **0.911** |  |  |
| **GaLog** | 0.096661 | **0.979** |  |  |
| **GdLog** | 0.153849 | **0.854** |  |  |
| HgLog | **0.651988** | -0.040 |  |  |
| **LaLog** | 0.094107 | **0.534** |  |  |
| **MnLog** | -0.11327 | **0.336** |  |  |
| **MoLog** | **0.370767** | **0.342** |  |  |
| **NdLog** | 0.025614 | **0.898** |  |  |
| **NiLog** | **0.339705** | **0.773** | **0.670** |  |
| PbLog | **0.556154** | 0.256 |  |  |
| **PrLog** | 0.013034 | **0.895** |  |  |
| RbLog | 0.010449 | -0.022 |  |  |
| SbLog | **0.437219** | 0.096 |  |  |
| SeLog | 0.085096 | 0.045 |  |  |
| **SmLog** | 0.038492 | **0.894** |  |  |
| SrLog | -0.07352 | -0.147 |  |  |
| **TlLog** | 0.093255 | **0.436** |  |  |
| **TmLog** | 0.123442 | **0.901** |  |  |
| **ULog** | 0.105618 | **0.740** | 0.134 |  |
| **VLog** | 0.101888 | **0.916** |  |  |
| **YbLog** | 0.110439 | **0.911** |  |  |
| ZnLog | **0.529369** | 0.186 |  |  |
